# Common modelling assumptions affect the joint moments measured during passive joint mobilizations

**DOI:** 10.1038/s41598-023-44576-8

**Published:** 2023-10-18

**Authors:** Axel Koussou, Raphaël Dumas, Eric Desailly

**Affiliations:** 1 Fondation Ellen Poidatz, Pôle Recherche and Innovation, 77310 Saint-Fargeau-Ponthierry, France; 2 Univ Lyon, Univ Gustave Eiffel, Univ Claude Bernard Lyon 1, LBMC UMR T9406, 69622 Lyon, France

**Keywords:** Health care, Medical research

## Abstract

Joint resistance to passive mobilization has already been estimated in-vivo in several studies by measuring the applied forces and moments while manipulating the joint. Nevertheless, in most of the studies, simplified modelling approaches are used to calculate this joint resistance. The impact of these simplifications is still unknown. We propose a protocol that enables a reference 3D inverse dynamics approach to be implemented and compared to common simplified approaches. Eight typically developed children and eight children with cerebral palsy were recruited and underwent a passive testing protocol, while applied forces and moments were measured through a 3D handheld dynamometer, simultaneously to its 3D kinematics and the 3D kinematics of the different segments. Then, passive joint resistance was estimated using the reference 3D inverse dynamics approach and according to 5 simplified approaches found in the literature, i.e. ignoring either the dynamometer kinematics, the measured moments alone or together with the measured tangential forces, the gravity and the inertia of the different segments, or the distal segments kinematics. These simplifications lead to non-negligible differences with respect to the reference 3D inverse dynamics, from 3 to 32% for the ankle, 4 to 34% for the knee and 1 to 58% for the hip depending of the different simplifications. Finally, we recommend a complete 3D kinematics and dynamics modelling to estimate the joint resistance to passive mobilization.

## Introduction

Human joints are complex mechanisms made of a set of anatomical structures allowing their proper functioning and preservation. The characterization of a musculo-articular complex, including all the anatomical structures spanning the joint, requires the measurement of its resistance to mobilization. This measure enables the mechanical properties of interest such as joint stiffness or musculo-tendon parameters such as muscle slack length^1,2^ to be determined. In neurological pathologies, for instance in children with cerebral palsy, a hyper-resistance to stretch may result from several causes being either neural or non-neural^3^. Being able to quantitatively assess this hyper-resistance to stretch as accurately as possible may help to better define a subject-specific treatment. However, the current clinical evaluations of this hyper-resistance have been criticized, among other reasons, for their poor reliability^4–7^. Objective, quantitative and robust measurements are therefore crucial for the accurate evaluation of the joint resistance to stretch and treatment efficacy^4^.

Thus, the instrumentation of the evaluation of joint resistance to mobilization has recently been studied more intensively. Through inverse dynamics computations, several protocols have been proposed to estimate, in vivo, the joint resistance to motion by measuring the applied forces and moments while manipulating the joint^8–16^, using handheld dynamometers, which are practical and easy to use. The method requires to model the biomechanical system by defining its geometry, joints linkage, inertial properties and to measure the 3D kinematics as well as the external forces and moments applied to the segments. The level of complexity of those chosen models are variable in the literature. In most of the studies, some important modelling assumptions and simplifications are used. Five main assumptions are commonly found in the literature. First, some authors consider that the axes of the dynamometer are aligned with those of the manipulated segment^8,9,11–16^. This way, the kinematics of the dynamometer is not required. However, the direction of the forces and moments applied to the segment is not correctly modelled, which can lead to an approximation of the passive joint resistance. Similarly, some authors use simpler dynamometers, ignoring some components of the measured forces and moments, approximating therefore the correct amount of resistance^9,11,14,15^. Moreover, for small and light segments as the foot, inertia and gravity is sometimes ignored in some studies^8,9, 13,16^. We wonder if these assumptions are valid, especially during high velocities mobilizations where inertial effects can be non-negligible. Finally, some authors assume that during the mobilization, distal segments are fixed with respect to the solicited one^8,9,14,16^, neglecting dynamics of the distal segments, which have their own kinematics.

All these simplifying hypotheses are made for practical reasons (cost, ease of use…). Nevertheless, the effect of those simplifications on the passive joint resistance remains unknown. The evaluation of the amount of approximations caused by each of these assumptions is therefore needed. For this purpose, a protocol that enables a reference 3D inverse dynamics approach has to be developed with the biomechanical modelling of the different segments of the lower limb with their inertial properties, as well as the measure of all the 3D forces and moments applied.

The aim of this work is to assess the impact of the modelling assumptions on the joint moments computed by inverse dynamics during joint mobilization.

## Method

### Subjects

Eight typically developed (TD) children (mean age: 11.64 ± 2.93; 5 males) and eight children with cerebral palsy (CP) (mean age: 13.37 ± 2.78; 5 males) were tested. TD children were included if they were between 7 and 18 years old, able to cooperate, and affiliated to the French social security system. They had no history of major orthopedic diagnosis or pain in the lower back, pelvis, or lower extremity. Inclusion criteria for the CP children were age between 7 and 18, diagnosed spastic CP, I to III levels on the Gross Motor Function Classification System, ability to cooperate, and affiliation to the French social security system. Exclusion criteria were any surgery or botulinum toxin injection within 6 months prior to the study, and diagnosed dystonia.

The protocol was approved by the ethical committee of the Comité de Protection des Personnes—Ouest IV. Voluntary adhesion of all the participants and informed consent of the legal representative were obtained. All methods were performed in accordance with the relevant guidelines and regulations.

### Experimental procedure

**Figure 1:**
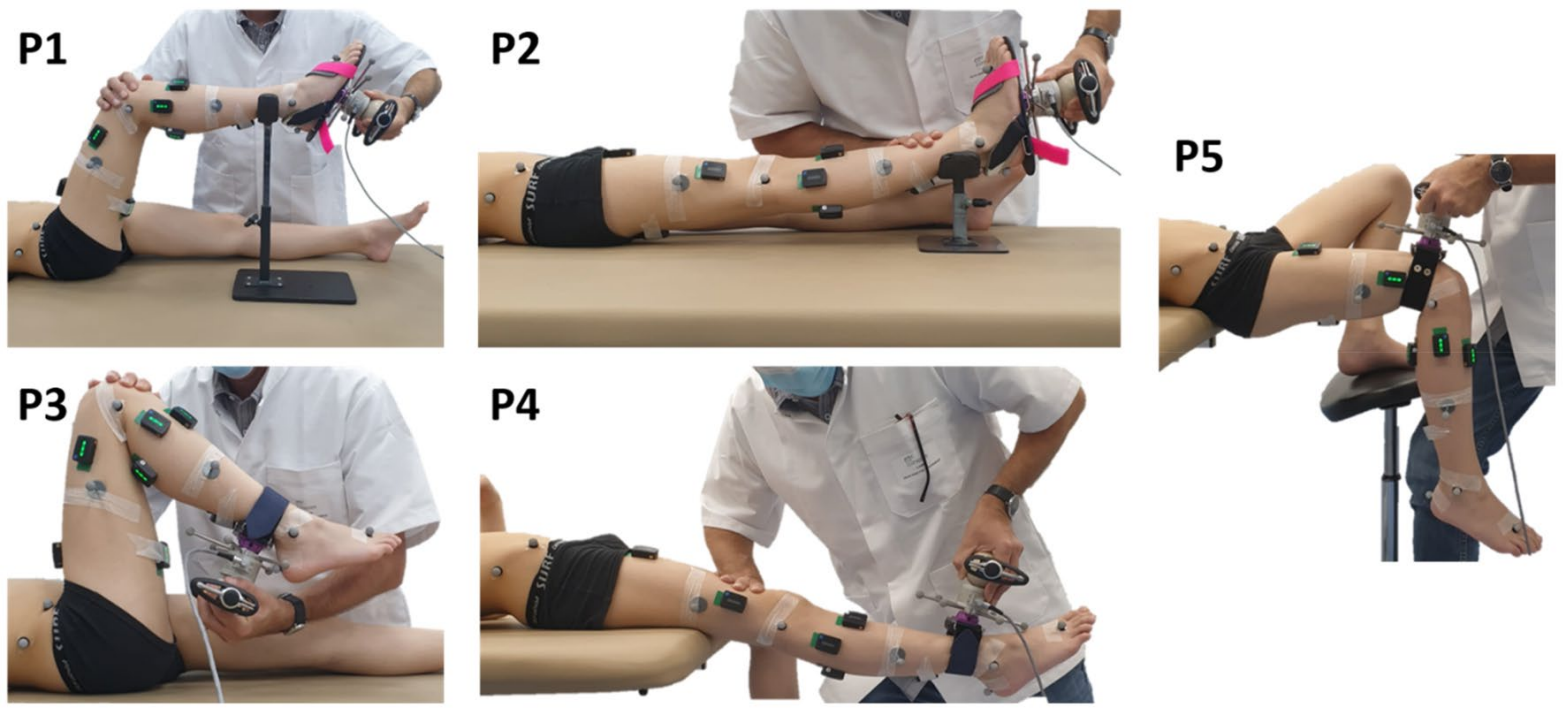
Test positions. Joints were mobilized through sagittal range of motion using a 3D handheld dynamometer. Dynamometer and body segment kinematics was measured with reflective markers and motion analysis system. Muscle activity was controlled with surface electromyography.

A passive testing protocol was designed to obtain continuous joint angle and joint resistance measurements while the subject’s joint was manipulated using a 3D handheld dynamometer (Sensix, Poitiers, FR, 2000 Hz), measuring triaxial applied forces and moments, through full available sagittal range of motion (ROM) (Fig. 1). Three joints were mobilized three times in five different positions (P1 to P5) at low (LV) and high (HV) velocity. It is important to note that only the stretch of the antagonist muscles is made at high velocity, the return to the starting position is made slowly:

- Ankle with the knee at 90° and 0° (positions P1, P2)
- Knee with the hip at 90° and 0° (positions P3, P4)
- Hip with free knee only at LV (position P5)

During all stretches, 3D kinematics of the pelvis and lower limbs was determined using a marker set, placed over specific body landmarks (lower limb Plug-in-Gait marker set with two additional markers placed over the iliac bone to take into account that the posterior superior iliac markers are not visible when the patient is lying) and a motion analysis system (15 cameras VICON, Oxford, UK, 100 Hz). Moreover, six markers, rigidly attached to the dynamometer, were used to track its position and orientation. Dynamic data (dynamometer forces and moments) were synchronously recorded and then downsampled at motion frequency.

EMG activities were synchronously recorded using pre-amplified dual differential surface electrodes (DE-2.1, DelSys, Inc., Boston, USA, 2000 Hz) placed over the rectus femoris, vastus lateralis, semitendinosus, tibialis anterior, peroneus longus, soleus and gastrocnemius muscles. Electrode locations, determined by the same investigator for each subject, according to the Surface Electromyography for the Non-Invasive Assessment of Muscles (SENIAM) guidelines, were prepared by shaving the skin and cleaning with alcohol. EMG activities were measured to check muscle inactivity during low velocity stretches or the occurrence of muscle reflex during high velocity stretches.

### Joint resistance modelling approaches

#### Kinematics

A seven segment, 24 degrees-of-freedom model of the pelvis and lower extremities was used to characterize joint kinematics. Six degrees of freedom were used to define the position and orientation of the pelvis, three degrees of freedom were used to define each hip, knee or ankle joint.

Body segment coordinate systems, joint centers and segment lengths were established using the marker positions collected during a calibration trial. For each segment ${\varvec{i}}$, a reference frame and the homogeneous matrix, ${\mathbf{T}}_{0,{\varvec{i}}}$, defining both the orientation (${\mathbf{R}}_{0,{\varvec{i}}}$) and the position $({\mathbf{P}}_{0,{\varvec{i}}}$) of the segment reference frame with respect to the global reference frame, was determined. Then, joint angles were calculated using the Cardan angles.

In the same way, using dynamometer marker positions, a dynamometer reference frame and its homogeneous matrix,${\mathbf{T}}_{0,{\varvec{D}}{\varvec{y}}{\varvec{n}}}$, was determined.

#### Inverse dynamics

**Figure 2:**
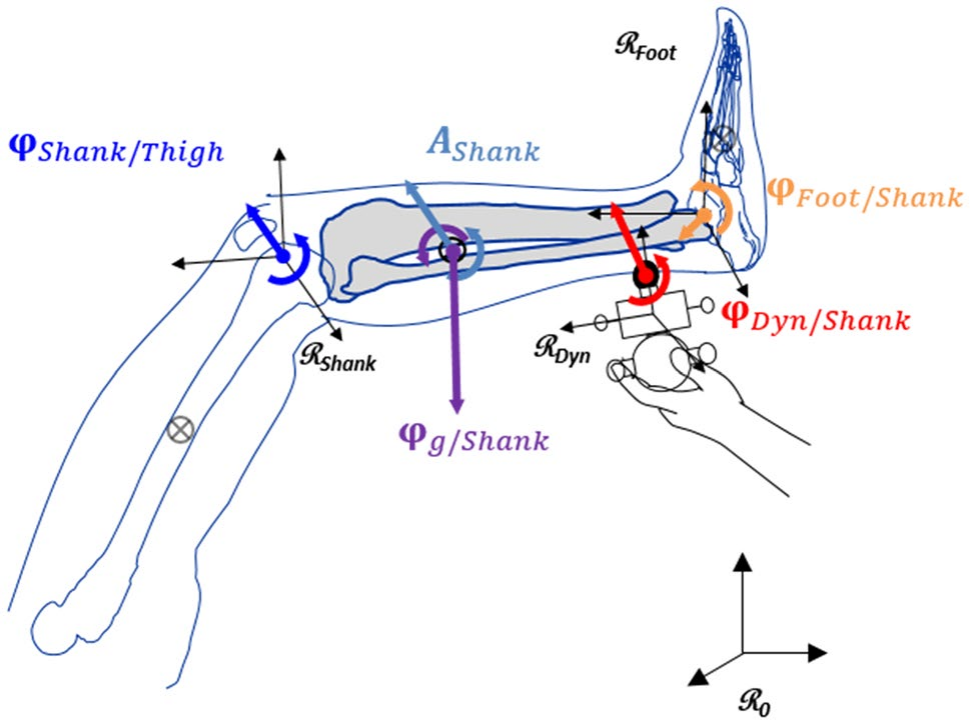
Free body diagram of the position P3 test. The joint resistance of the knee ${\mathbf{\varphi }}_{\mathrm{Shank}/\mathrm{Thigh}}$ is calculated by applying Eq. (9) to the shank segment. ${\mathbf{A}}_{\mathrm{Shank}}$ represents the inertial action matrix and is computed from the subject’s shank inertial parameters and kinematics. ${\mathbf{\varphi }}_{\mathrm{g}/\mathrm{Shank}}$ is the weight action matrix. ${\mathbf{\varphi }}_{\mathrm{Dyn}/\mathrm{Shank}}$ represents the measured forces and moments applied to the shank by the handheld dynamometer accordingly to their kinematics. ${\mathbf{\varphi }}_{\mathrm{Foot}/\mathrm{Shank}}$ represents the forces of the foot on the shank segment computed with Eq. (10).

Inverse dynamic analysis, using homogeneous matrix, was used to compute the hip, knee, and ankle joint moments^17,18^. A free body diagram example for the position P3 is proposed in Fig. 2. The convention used to represent the curves of moments corresponds to that recommended by the International Society of Biomechanics^19^. The reader can invert the ankle and hip curves to find a convention more generally used by the clinicians.

First, measured forces and moments from the handheld dynamometer, expressed in its reference frame, form the matrix:

1 $${\mathbf{\varphi }}_{{Dyn_{{R_{Dyn} }} }} = { }\left[ {\begin{array}{*{20}c} 0 & {\quad - M_{z} } & {\quad M_{y} } & {\quad F_{x} } \\ {M_{z} } & {\quad 0} & {\quad - M_{x} } & {\quad F_{y} } \\ { - M_{y} } & {\quad M_{x} } & {\quad 0} & {\quad F_{z} } \\ { - F_{x} } & {\quad - F_{y} } & {\quad - F_{z} } & {\quad 0} \\ \end{array} } \right]$$

where ${F}_{x}$, ${F}_{y}$, ${F}_{z}$ are the components of forces and ${M}_{x}$,${M}_{y}$,${M}_{z}$ are the components of moments.

Then, this tensor is expressed in the global reference frame thanks to the dynamometer transformation matrix:

2 $${\mathbf{\varphi }}_{{Dyn_{R0} }} = { }{\mathbf{T}}_{0,Dyn} \cdot {\mathbf{\varphi }}_{{Dyn_{RDyn} }} \cdot {\mathbf{T}}_{0,Dyn}^{t}$$

where superscript t stands for matrix transpose.

The gravity acceleration matrix is given, in the global reference frame, by:

3 $${\mathbf{H}}_{{g_{R0} }} = { }\left[ {\begin{array}{*{20}c} 0 & {\quad 0} & {\quad 0} & {\quad 0} \\ 0 & {\quad 0} & {\quad 0} & {\quad 0} \\ 0 & {\quad 0} & {\quad 0} & {\quad - 9.81} \\ 0 & {\quad 0} & {\quad 0} & {\quad 0} \\ \end{array} } \right]$$

Finally, subject’s body segment inertial parameters were estimated using the regression equations developed for children by Jensen^20^. Segment pseudoinertial matrices were determined thanks to the parallel axis theorem and expressed in the segment reference frame:

4 $${\mathbf{J}}_{Ri} = { }\left[ {\begin{array}{*{20}c} {\left[ {{\mathbf{K}}_{O} } \right]} & {\quad \left[ {\mathbf{q}} \right]} \\ {\left[ {{\mathbf{q}}^{t} } \right]} & {\quad m} \\ \end{array} } \right]$$

where $\left[{\mathbf{K}}_{O}\right]$, $\left[{\varvec{q}}\right]$ and *m* are, respectively, the segment Poinsot inertia matrix, first moment of inertia (product between the mass and the position of center of mass with respect to the reference frame origin) and mass.

Thus, through an iterative process, as described in the following algorithm, the joint moments (joint resistance in the present case), ${\mathbf{\varphi }}_{{i/i+1}_{Ri}}$, are obtained, at every instant, in their local frame.

$${Initialize\,\, \mathbf{\varphi }}_{{0/1}_{R0}}=0$$

$$For i=1 \left(foot\right) :3 \left(thigh\right)$$

5 $${\mathbf{H}}_{0,i} = {\ddot{\mathbf{T}}}_{0,i} \cdot T_{0,i}^{ - 1}$$

6 $${\mathbf{J}}_{{i_{R0} }} = {\mathbf{T}}_{0,i} \cdot {\mathbf{J}}_{{i_{Ri} }} \cdot {\mathbf{T}}_{0,i}^{t}$$

7 $${\mathbf{A}}_{{i_{R0} }} = {\mathbf{H}}_{0,i} \cdot {\mathbf{J}}_{{i_{R0} }} - {\mathbf{J}}_{{i_{R0} }} \cdot {\mathbf{H}}_{0,i}^{t}$$

8 $${\boldsymbol{\varphi }}_{{g/i_{R0} }} = {\mathbf{H}}_{{g_{R0} }} \cdot {\mathbf{J}}_{{i_{R0} }} - {\mathbf{J}}_{{i_{R0} }} \cdot {\mathbf{H}}_{{g_{R0} }}^{t}$$

*If i is the mobilized segment*

9 $${\varvec{\varphi }}_{{i/i + 1_{R0} }} = - {\varvec{A}}_{{i_{R0} }} + {\varvec{\varphi }}_{{g/i_{R0} }} + {\varvec{\varphi }}_{{i - 1/i_{R0} }} + {\varvec{\varphi }}_{{Dyn/i_{R0} }}$$

*Else*

10 $${\varvec{\varphi }}_{{i/i + 1_{{R0}} }} = ~ - ~{\mathbf{A}}_{{i_{{R0}} }} + ~{\varvec{\varphi }}_{{g/i_{{R0}} }} + ~{\varvec{\varphi }}_{{i - 1/i_{{R0}} }}$$

*End*

11 $${\varvec{\varphi }}_{{i/i + 1_{Ri} }} = {\mathbf{T}}_{0,i}^{ - 1} \cdot {\varvec{\varphi }}_{{i/i + 1_{R0} }} \cdot {\mathbf{T}}_{0,i}^{{ - 1^{t} }}$$

*End*

This inverse dynamic process with a complete 3D modelling of the joints and the dynamometer was considered as our reference. According to the literature, several modelling assumptions and simplifications were studied.

##### Dynamometer kinematics

We considered that the dynamometer kinematics was the same as the segment on which it was attached (Case A). Then, ${\mathbf{T}}_{0,Dyn}$ was modified. Its orientation part was supposed the same as the solicited segment, *i*, and its position was assumed fixed in the segment frame.

12 $${\mathbf{T}}_{0,Dyn} = { }\left[ {\begin{array}{*{20}c} {\left[ {{\mathbf{R}}_{0,Dyn} } \right]} & {\quad \left[ {{\mathbf{P}}_{0,Dyn} } \right]} \\ {0{ }0{ }0} & {\quad 1} \\ \end{array} } \right]{ } \to { }\left[ {\begin{array}{*{20}c} {\left[ {{\mathbf{R}}_{0,i} } \right]} & {\quad \left[ {{\varvec{P}}_{0,i} + {\varvec{R}}_{0,i} \overline{\user2{P}}_{i,Dyn} } \right]} \\ {0{ }0{ }0} & {\quad 1} \\ \end{array} } \right]$$

with ${\overline{\mathbf{P}} }_{i,Dyn}$ the averaged position of the dynamometer in the *i*-th segment reference frame.

Thus, this simplification will mainly affect the expression of the dynamometer forces and moments in the global reference frame.

##### Measured forces and moments

We ignored some components of the measured forces and moments^9,11,14,15^. First, the measured moments were considered as null (Case B).

13 $${\mathbf{\varphi }}_{{Dyn_{RDyn} }} = { }\left[ {\begin{array}{*{20}c} 0 & {\quad - M_{z} } & {\quad M_{y} } & {\quad F_{x} } \\ {M_{z} } & {\quad 0} & {\quad - M_{x} } & {\quad F_{y} } \\ { - M_{y} } & {\quad M_{x} } & {\quad 0} & {\quad F_{z} } \\ { - F_{x} } & {\quad - F_{y} } & {\quad - F_{z} } & {\quad 0} \\ \end{array} } \right]{ } \to { }\left[ {\begin{array}{*{20}c} {\quad 0} & {\quad 0} & {\quad 0} & {\quad F_{x} } \\ {\quad 0} & {\quad 0} & {\quad 0} & {\quad F_{y} } \\ {\quad 0} & {\quad 0} & {\quad 0} & {\quad F_{z} } \\ {\quad - F_{x} } & {\quad - F_{y} } & {\quad - F_{z} } & {\quad 0} \\ \end{array} } \right]$$

Second, only the main force of solicitation, as if we had a monoaxial load cell, was considered (Case C).

14 $${\mathbf{\varphi }}_{{Dyn_{RDyn} }} = \left[ {\begin{array}{*{20}c} 0 & {\quad - M_{z} } & {\quad M_{y} } & {\quad F_{x} } \\ {M_{z} } & {\quad 0} & {\quad - M_{x} } & {\quad F_{y} } \\ { - M_{y} } & {\quad M_{x} } & {\quad 0} & {\quad F_{z} } \\ { - F_{x} } & {\quad - F_{y} } & {\quad - F_{z} } & {\quad 0} \\ \end{array} } \right] \to \left[ {\begin{array}{*{20}c} 0 & {\quad 0} & {\quad 0} & {\quad 0} \\ 0 & {\quad 0} & {\quad 0} & {\quad 0} \\ 0 & {\quad 0} & {\quad 0} & {\quad F_{z} } \\ 0 & {\quad 0} & {\quad - F_{z} } & {\quad 0} \\ \end{array} } \right]$$

##### Inertia and gravity

Inertia and gravity of the segment were supposed null^8,9,13^ (Case D).

15 $${\mathbf{A}}_{{i_{R0} }} = 0 $$

16 $${\mathbf{\varphi }}_{{gi_{R0} }} = 0$$

##### Distal segments movement

For the knee and hip tests, we studied the effect of considering that the distal segments (foot or foot and knee) are fixed relatively to the tested segment. To do so, distal segments kinematics was supposed the same as the tested joint (Case E).

Thus, for the knee joint tests (positions P3 and P4):

17 $${\mathbf{T}}_{0,ankle} = { }\left[ {\begin{array}{*{20}c} {\left[ {{\mathbf{R}}_{0,ankle} } \right]} & {\quad \left[ {{\mathbf{P}}_{0,ankle} } \right]} \\ {0{ }0{ }0} & {\quad 1} \\ \end{array} } \right]{ } \to { }\left[ {\begin{array}{*{20}c} {\left[ {{\mathbf{R}}_{0,knee} } \right]} & {\quad \left[ {{\varvec{P}}_{0,knee} + {\varvec{R}}_{0,knee} \overline{\user2{P}}_{knee,ankle} } \right]} \\ {0{ }0{ }0} & {\quad 1} \\ \end{array} } \right]$$

with ${\overline{\mathbf{P}} }_{knee/ankle}$ is the averaged position of the ankle in the knee reference frame and for the hip joint test (position P5), we have:

18 $${\mathbf{T}}_{0,knee} = \left[ {\begin{array}{*{20}c} {\left[ {{\mathbf{R}}_{0,knee} } \right]} & {\quad \left[ {{\mathbf{P}}_{0,knee} } \right]} \\ {0 0 0} & {\quad 1} \\ \end{array} } \right] \to \left[ {\begin{array}{*{20}c} {\left[ {{\mathbf{R}}_{0,hip} } \right]} & {\quad \left[ {{\varvec{P}}_{0,hip} + {\varvec{R}}_{0,hip} \overline{\user2{P}}_{hip,knee} } \right]} \\ {0 0 0} & {\quad 1} \\ \end{array} } \right]$$

19 $${\mathbf{T}}_{0,ankle} = \left[ {\begin{array}{*{20}c} {\left[ {{\mathbf{R}}_{0,ankle} } \right]} & {\quad \left[ {{\mathbf{P}}_{0,ankle} } \right]} \\ {0 0 0} & {\quad 1} \\ \end{array} } \right] \to \left[ {\begin{array}{*{20}c} {\left[ {{\mathbf{R}}_{0,hip} } \right]} & {\quad \left[ {{\varvec{P}}_{0,hip} + {\varvec{R}}_{0,hip} \overline{\user2{P}}_{hip,ankle} } \right]} \\ {0 0 0} & {\quad 1} \\ \end{array} } \right]$$

with ${\overline{\mathbf{P}} }_{hip/ankle}$ and ${\overline{\mathbf{P}} }_{hip/knee}$ are the averaged positions of, respectively, the ankle and the knee joints in the hip reference frame.

### Statistics

We evaluated the different simplifications by calculating the difference between the joint moments computed through the complete 3D inverse dynamics approach and the other methods at minimal and maximal angles (M_θMin_ and M_θMax_ respectively). With non-normal data (verified by Shapiro–Wilk test), M_θMin_ and M_θMax_ of the reference inverse dynamics method were compared at those of the different cases, pooling TD and CP children and pooling all velocities (except for Case D), with non-parametric Wilcoxon signed-rank tests for paired samples. The level of significance for the tests was *p* = 0.05/5 = 0.01 considering Bonferroni correction for multiple comparisons. Confidence intervals were calculated according to Campbell and Gardner^21^.

In order to estimate the amount of approximation made with the different cases over the entire tested ROM, root-mean-square differences (RMSD) were calculated in respect to the reference inverse dynamics. RMSD was also expressed as a percentage of the absolute maximum joint moment value (%Max). Correlation coefficients between reference inverse dynamics and the different simplification cases were also calculated. Inverse dynamics (Eqs. 1–19) and statistics calculation were implemented in Matlab 2020a (MathWorks, Natick, USA).

## Results

**Figure 3:**
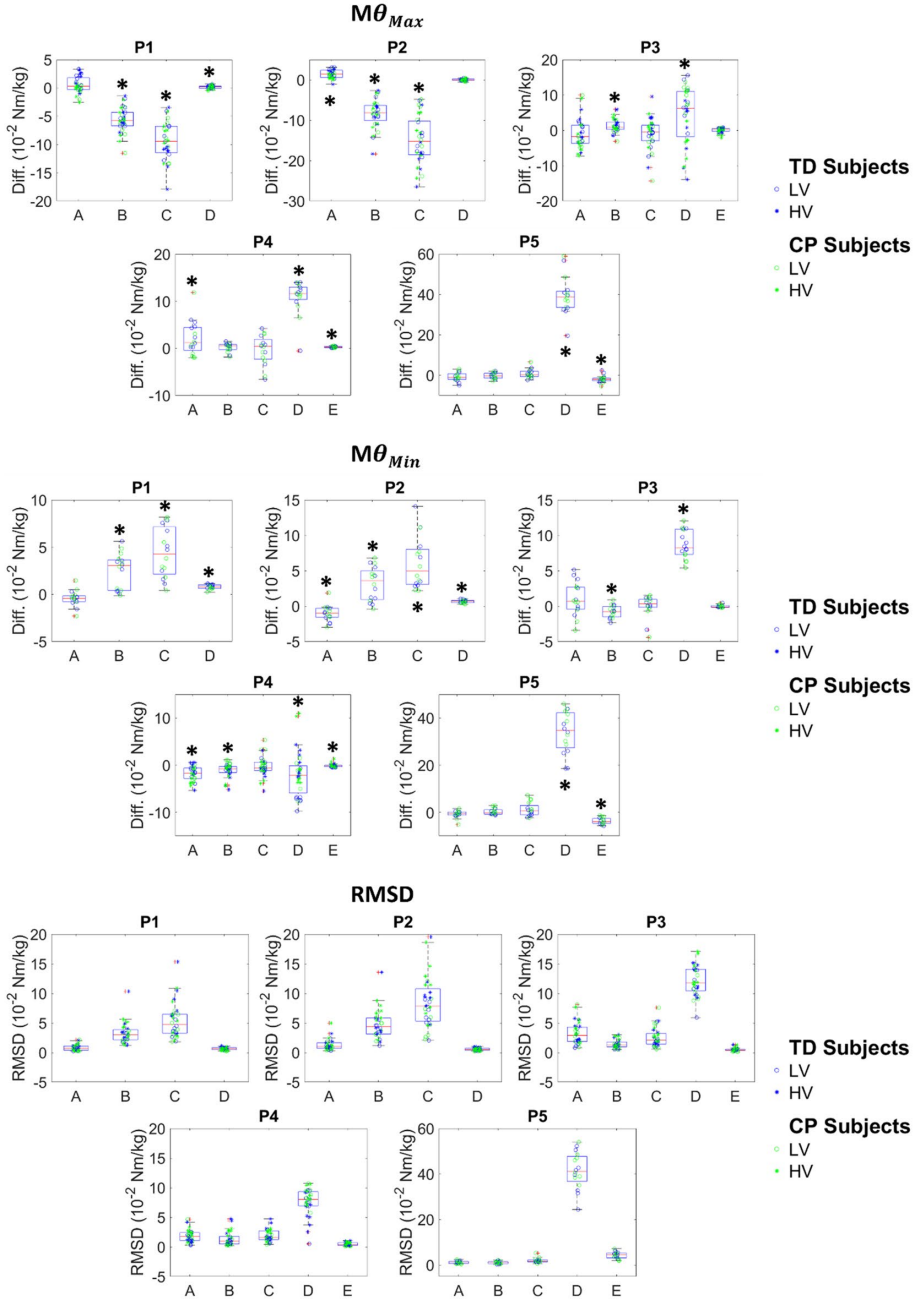
M_θMax_, M_θMin_ and RMSD differences (10^−2^ Nm/kg) of healthy and pathological subjects obtained for the different studied cases in all the positions. Asterisks indicate statically significant differences with the reference 3D inverse dynamics approach.

**Figure 4:**
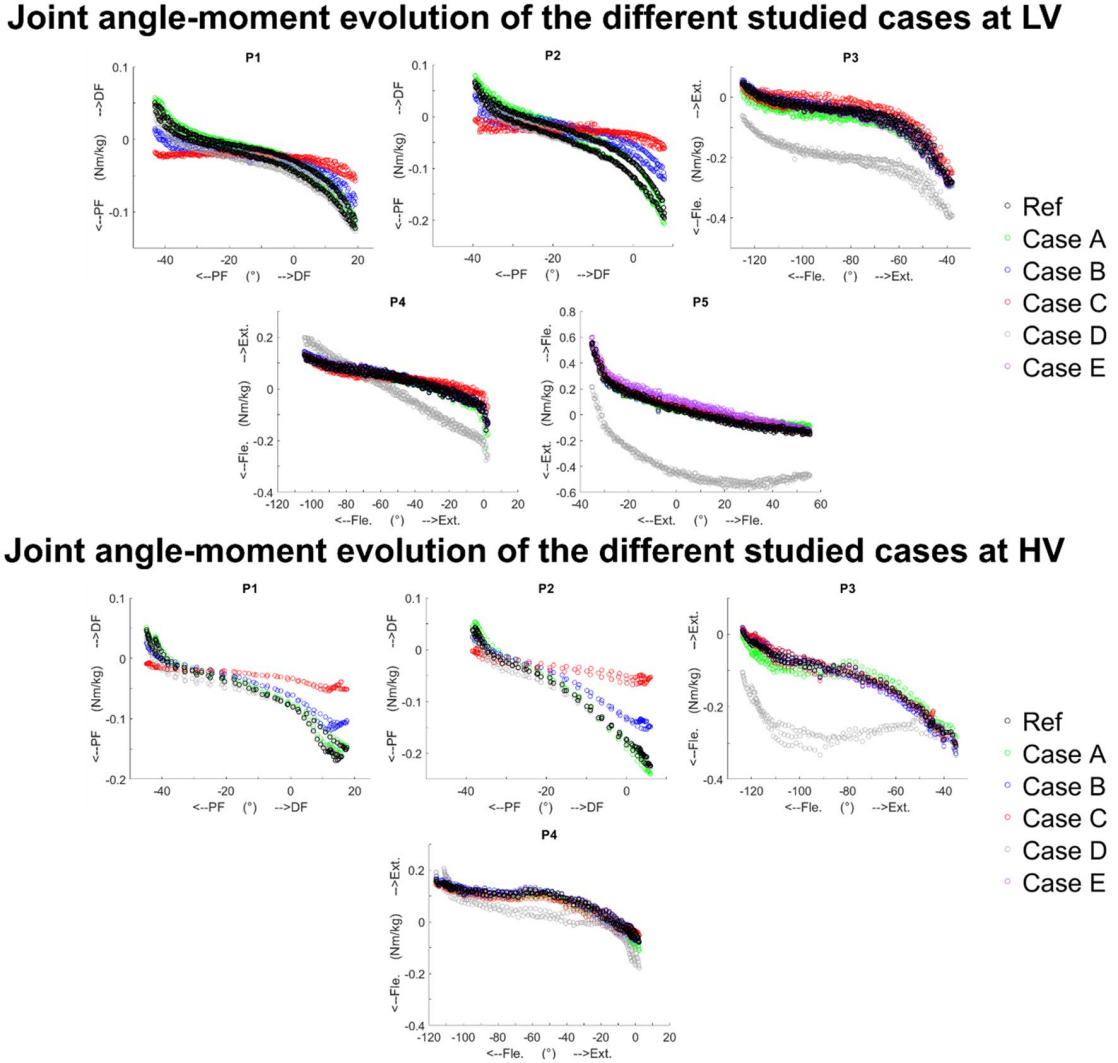
Relationships between the joint angle and moment of the different studied cases at low (LV) and high (HV) velocity for all the positions for one typically developed child.

Differences in M_θMin_ and M_θMax_ and RMSD for all cases and positions are presented in Figs. 3 and 4. Figure 4 represents the relationships between the joint angle and the joint moment. Before pooling the results, although most CP patients demonstrated muscle reflex at HV, no significant differences, in the amount of joint moment approximation caused by the simplifications, according to pathology or velocity were found. Conversely, significant differences between the reference inverse dynamics and the different cases (pooling all results) were found. Results are presented below for each case considered. Detailed results are available in supplementary material.

### Dynamometer kinematics (Case A)

Significant differences with respect to the reference inverse dynamics method were found on M_θMax_ for positions P2 and P4 (median differences [IQR] of 0.8 [0.1–2.2] and 1.2 [− 0.4–4.4] 10^−2^ Nm/kg respectively). The *p* value was also low (p-value: 0.06) for position P3 with median difference [IQR] of − 1.8 [− 3.7–1.4] 10^−2^ Nm/kg. Significant differences were found on M_θMin_ for positions P2 and P4 (median differences [IQR] of − 1.0 [− 1.6 to − 0.3] and − 1.7 [− 2.8 to − 0.6] 10^−2^ Nm/kg respectively). Again *p* value was low (*p* value: 0.09) for position P3 with median difference [IQR] of 0.7 [− 0.4–2.7] 10^−2^Nm/kg. Thus, M_θMin_ at the knee joint is likely to be slightly underestimated at position P3 and overestimated at position P4 (Figs. 3, 4) whereas M_θMax_ is likely to be slightly underestimated at position P4 (Figs. 3, 4).

RMSD values lead to a median approximation up to 7.6% of the absolute maximum joint resistance value measured. Consistency of the method relative to reference inverse dynamics was verified with high coefficient of correlation values (R > 0.98).

### Measured forces and moments (Cases B-C)

Measured forces and moments are available in the Supplementary Material—Fig. S1. When measured moments are ignored (Case B), significant differences with respect to the reference inverse dynamics method were found on M_θMax_ for positions P1, P2 and P3 (median differences [IQR] of − 5.8 [− 6.7 to − 4.3], − 6.1 [− 8.4 to − 4.7] and 1.0 [0.3–2.3] 10^−2^ Nm/kg respectively), and on M_θMin_ for positions P1, P2, P3 and P4 (median differences [IQR] of 3.0 [0.4–3.6], 3.6 [0.9–5.0], − 0.8 [− 1.5–0.0] and − 0.8 [− 1.6 to − 0.3] 10^−2^ Nm/kg respectively).

When measured moments are ignored (Case B), RMSD values are important for the ankle position test (positions P1 and P2) leading to a median approximation up to 17% of the absolute maximum joint resistance value measured. Consistency of the method relatively to reference inverse dynamics was verified with high coefficient of correlation values (R > 0.98).

When we consider only a monoaxial load (Case C), significant differences with respect to the reference inverse dynamics method were found on M_θMax_ for positions P1 and P2 (median differences [IQR] of − 9.4 [− 11.5 to − 6.8] and − 11.0 [− 15.7 to − 7.6] 10^−2^ Nm/kg respectively), and on M_θMin_ for positions P1 and P2 (median differences [IQR] of 4.3 [2.1–7.1] and 4.9 [3.1–8.0] 10^−2^ Nm/kg respectively).

When we consider only a monoaxial load (Case C), RMSD values increase and remain important for P1 and P2 and become non-negligible for P3 and P4 (%Max > 5%). Consistency of the method relatively to reference inverse dynamics remains verified with high coefficient of correlation values (R > 0.92). However, for some trials in positions P1 and P2, correlation was lower (0.4 < R < 0.9).

For both cases B and C, M_θMax_ at the ankle joint is likely to be overestimated and M_θMin_ slightly underestimated at positions P1 and P2 (Figs. 3, 4).

### Inertia and gravity (Case D)

Significant differences with respect to the reference inverse dynamics method were found on M_θMax_ for positions P1, P3, P4 and P5 (median differences [IQR] of 0.2 [− 0.1–0.3], 6.2 [1.8–11.0], 11.6 [10.4–13.0] and 38.7 [33.5–41.5] 10^−2^ Nm/kg respectively), and on M_θMin_ for positions P1, P2, P3, P4 and P5 (median differences [IQR] of 0.8 [0.6–1.0], 0.7 [0.5–0.8], 8.2 [7.3–10.9], − 2.2 [− 5.9 to − 0.1] and 34.7 [27.4–42.2] 10^−2^ Nm/kg respectively). M_θMax_ and M_θMin_ at the knee joint are likely to be underestimated at position P3 (Figs. 3, 4). M_θMax_ is likely to be also underestimated at position 4 whereas results are more variable concerning M_θMin_. M_θMax_ and M_θMin_ at the hip joint are likely to be underestimated at position P5.

Ignoring inertia and gravity leads to important RMSD for positions P3, P4 and P5 with median approximations between 30 and 57% of the absolute maximum joint resistance value measured. For this case, the impact of this modelling assumption seems affected by the movement velocity. At low velocity, consistency of the method relatively to reference inverse dynamics remains verified (R > 0.98) but at high velocity the correlation decreases for positions P3 and P4 (R of 0.76 [0.54–0.89] and 0.83 [0.64–0.87], respectively).

### Distal segments movement (Case E)

Significant differences with respect to the reference inverse dynamics method were found on M_θMax_ for positions P4 and P5 (median differences [IQR] of 0.3 [0.1–0.4] and − 2.1 [− 2.8 to − 1.4] 10^−2^ Nm/kg respectively), and on M_θMin_ for positions P4 and P5 (median differences [IQR] of − 0.2 [− 0.3–0.0] and − 4.0 [− 4.6 to − 2.6] respectively). M_θMax_ and M_θMin_ at the hip joint are likely to be overestimated at position P5 (Figs. 3, 4).

In this case, low RMSD values were found for positions P3 and P4 with mean %Max inferior to 2.0%. For position P5, RMSD values were more important and lead to a mean approximation up to 6% of the maximum joint resistance value measured. Consistency of the method relative to reference inverse dynamics was verified with high coefficient of correlation values (R > 0.99).

## Discussion

This study aimed to evaluate how different modelling assumptions that can be found in the literature affect the computation of the joint resistance to mobilization. To do so, we proposed a protocol that enabled a reference inverse dynamics approach to be implemented. Applied forces and moments were measured through a 3D handheld dynamometer, simultaneously to its 3D kinematics and the 3D kinematics of the different segments.

Significant differences have been found between the reference inverse dynamics and the different simplifications studied for the joint moment at minimal and maximal angles (M_θMin_ and M_θMax_). However, the discussion will be focused on simplifications that led to the approximations that would impact the evaluation of joint resistance.

### Oversimplified modelling assumptions

Measuring the dynamometer kinematics is rarely done in the literature. Instead, its kinematics is supposed to be the same as the segment on which it is attached (Case A), assuming that the axes of the dynamometer are aligned with those of the manipulated segment^8,9,12,14^. However, this simplification leads to approximations on the orientation and position of the measured forces and moments, which cause differences with respect to the reference inverse dynamics method. Particularly, it was the case during the knee stretches (positions P3 and P4), where RMSD values or median differences on M_θMax_ and M_θMin_ are the higher. Indeed, in these test conditions, applying a movement profile to the dynamometer that enables to maintain a constant position and orientation of the dynamometer in the shank reference frame while mobilizing the knee joint seems more complex. Simplifying the dynamometer kinematics could lead to a slight underestimation or overestimation of the maximal knee extension moment at positions P3 and P4 respectively, and a slight overestimation of the maximal knee flexion moment at position P4 only [up to a median difference of 6% of the reference absolute maximal joint moment in position P3].

Some authors do not consider measured moments or use monoaxial load cells^9,11,14,15^, whereas mobilizations are not purely in the sagittal plane (Supplementary Material—Fig. S1). Interestingly, ignoring the measured moments alone or also the tangential measured forces components leads to non-negligible differences for the ankle stretches (positions P1 and P2). For other positions, results seems to be more variable with some trials showing important differences with the reference inverse dynamics method. These results show that when applying a stretch, the evaluator does not apply a purely monoaxial force. This is especially true for ankle stretches, where both the measured forces and moments have a great impact. Thus, Case B and Case C enhance the importance of using a 3D dynamometer. Not measuring the full tensor underestimate the maximum plantarflexion and dorsiflexion moments [up to a median difference of 63% of the reference absolute maximal joint moment in position P2 for Case C].

Ignoring the inertia and the gravity of the segments (Case D) is a well-known effect in the literature^22–24^. Thus, in the literature, inertia and gravity are more frequently ignored for small segments (i.e. the foot) than for larger segments. This study confirms that this simplification is acceptable for the ankle stretches, where small differences with the reference are found, but not for the knee and hip stretches. This is in agreement with what is done in the literature, where this simplification is made only for the ankle stretches^8^. Moreover, it is interesting to see the effect of the velocity on the resulting approximation for the knee stretches (positions P3 and P4). Indeed, differences on M_θMax_ and M_θMin_ were greater at high velocity, and similarly, consistency with the reference inverse dynamics method, was impacted, with mean R values decreasing importantly. The inertia and the gravity of the segments must be considered when computing knee or hip resistance to mobilizations [up to a median difference of 55% of the reference absolute maximal joint moment in position P5].

The last assumption tested consists of ignoring the kinematics of the distal segments (Case E). Authors assume that during the mobilization, distal segments are fixed with respect to the manipulated one, neglecting the inertial and gravitational efforts due to the distal segments, as they have their own kinematics. This simplification appears to be acceptable for the knee stretches (positions P3 and P4), which is in agreement with Bar-On et al.^8^ but starts to be non-negligible for hip stretches (P5), as shown by RMSD and M_θMax_ and M_θMin_ values. Measuring distal segment kinematics is recommended especially for hip assessments [up to a median difference of 7% of the reference absolute maximal joint moment in position P5].

Our results question the use of oversimplified approaches to compute joint resistance to mobilization. Certainly, some of the tested simplifications may remain arguable, justified by cost or time limitations. First, more trained evaluators might manage to apply a movement profile to the dynamometer that strictly follows the joint plane (Case A) or limits the non-axial forces and moments (Case B and C), reducing the differences with the reference inverse dynamics method. Thus, it would require a training to compensate the differences of the cases A, B or C. Nevertheless, reference data (based on dynamometer kinematics or 3D load repartition during the mobilization) would be necessary for such a training. Second, the 3D inverse dynamics approach could be considered more complex to implement. In a clinical context, time is a valuable resource. However, we did not evaluate if 3D inverse dynamics approach takes more time than other literature methods. It should also be noted that some of the proposed methods in the literature require the measurement of specific distances, i.e. distance between the dynamometer or lad cell and the joint center that could also be time-consuming^8,9^. Moreover, the biomechanical models used in the literature methods differ from the ones used in gait analysis, which can lead to approximations when comparing the joint moments measured during passive stretches and the ones during gait^25^. With the reference inverse dynamics method, the biomechanical model can be the one used in gait analysis (use of the same marker set).

Finally, it must be considered that differences in the joint resistance measure may lead to an altered estimation of mechanical properties of interest such as joint passive stiffness or musculo-tendon parameter such as muscle slack length^1,2^. For all these reasons, we recommend a complete kinematics and dynamics modelling appears before considering analyses for clinical purposes.

### Limitations

Various limitations are present in this study. First, more than 16 subjects could have been tested; this could have led to a greater homogeneity of the sample. However, both box-plots and non-parametric tests were able to identify confidently the effects of the tested simplifications. Second, simplifications have been studied separately, whereas in the literature, there are several examples where these ones have been combined. For example, in most of the studies dynamometer kinematics (Case A) and distal segments movement (Case E) are simultaneously ignored. Nevertheless, since inverse dynamics is a linear problem, we expect that the resulting approximations add cumulatively.

## Conclusion

Joint resistance to mobilization has been estimated in-vivo in several studies, through inverse dynamics computations, by measuring the applied forces and moments while manipulating the joint. Nevertheless, in most of the studies, simplified approaches are used to calculate joint moments. This study assessed the impact of these modelling assumptions.

Most of these assumptions lead to non-negligible differences with respect to a reference 3D inverse dynamics approach. Thus, literature results should be considered with caution. Finally, we recommend a complete kinematics and dynamics modelling before considering analyses for clinical purposes.

### Supplementary Information

Supplementary Information.

## Data Availability

All data generated or analysed during this study are included in this published article and its supplementary information files.
