# Supplementary material for: Common modelling assumptions affect the joint moments measured during passive joint mobilizations
Source: Sci Rep. 2023 Oct 18;13:17782. doi: 10.1038/s41598-023-44576-8 (PMC10584879; doi:10.1038/s41598-023-44576-8)

**Supplementary Material**

This supplementary material presents the results of measured intersegmental moments (Tables S1 and S2) and their difference (Tables S3-S8) of each considered simplified case with the reference inverse dynamics method at maximal angles (M_θMax_) and minimal angles (M_θMin_) detailed for each position and for each stretch velocity (low/LV or high/HV).

During HV test, only the stretch of the antagonist muscles is made at high velocity, the return to the starting position is made slowly. Thus, for this velocity results are presented only at the final stretching position, meaning:

- the maximum of dorsiflexion for positions P1 and P2
- the maximum of knee extension for position P3
- the minimum of knee extension for position P4

Root-mean-square differences between the reference inverse dynamics method and each considered simplified case, root-mean-square differences normalized by the absolute maximum joint moment, and coefficient of correlation are also presented for each velocity and position (Tables S9-S11).

Finally, the supplementary material S12 presents the experimental measured forces and moments during the different passive mobilizations.

**Table S1: Intersegmental moments at maximal angles for the different cases**

|  | **M_θMax_ (Nm/kg)**  **Median [IQR]** | | | | | | | | | | | | | | | | | |
| --- | --- | --- | --- | --- | --- | --- | --- | --- | --- | --- | --- | --- | --- | --- | --- | --- | --- | --- |
|  | Reference | | | Case A | | | Case B | | | Case C | | | Case D | | | Case E | | |
|  | LV &HV | LV | HV | LV &HV | LV | HV | LV &HV | LV | HV | LV &HV | LV | HV | LV &HV | LV | HV | LV &HV | LV | HV |
| P1 | -0.15  [-0.19--0.19] | -0.14  [-0.18--0.13] | -0.18  [-0.20--0.14] | -0.16  [-0.21--0.16] | -0.15  [-0.20--0.13] | -0.17  [-0.21--0.14] | -0.10  [-0.12--0.08] | -0.09  [-0.10--0.07] | -0.12  [-0.13--0.10] | -0.07  [-0.08--0.05] | -0.06  [-0.07--0.05] | -0.07  [-0.09--0.05] | -0.15  [-0.19--0.13] | -0.15  [-0.18--0.13] | -0.18  [-0.21--0.15] | N/A | N/A | N/A |
| P2 | -0.18  [-0.24--0.14] | -0.21  [-0.26--0.13] | -0.23  [-0.29--0.18] | -0.19  [-0.25--0.14] | -0.21  [-0.28--0.15] | -0.23  [-0.30--0.19] | -0.12  [-0.15--0.07] | -0.12  [-0.16--0.07] | -0.14  [-0.19--0.11] | -0.06  [-0.08--0.05] | -0.06  [-0.08--0.05] | -0.06  [-0.07--0.05] | -0.18  [-0.24--0.15] | -0.21  [-0.26--0.13] | -0.23  [-0.29--0.18] | N/A | N/A | N/A |
| P3 | -0.29  [-0.5--0.19] | -0.29  [-0.43--0.19] | -0.29  [-0.54--0.2] | -0.28  [-0.47--0.23] | -0.30  [-0.38--0.21] | -0.26  [-0.48--0.23] | -0.32  [-0.49--0.20] | -0.30  [-0.44--0.20] | -0.34  [-0.57--0.22] | -0.28  [-0.46--0.21] | -0.25  [-0.37--0.20] | -0.31  [-0.56--0.22] | -0.38  [-0.48--0.27] | -0.39  [-0.50--0.30] | -0.33  [-0.44--0.25] | -0.29  [-0.51--0.20] | -0.30  [-0.43--0.20] | -0.29  [-0.52--0.21] |
| P4 | N/A | -0.07  [-0.14--0.02] | N/A | N/A | -0.09  [-0.19--0.03] | N/A | N/A | -0.07  [-0.15--0.03] | N/A | N/A | -0.07  [-0.13--0.04] | N/A | N/A | -0.20  [-0.28--0.10] | N/A | N/A | -0.09  [-0.15--0.02] | N/A |
| P5 | N/A | 0.07  [-0.03-0.17] | N/A | N/A | 0.09  [0.02-0.17] | N/A | N/A | 0.06  [-0.02-0.17] | N/A | N/A | 0.05  [-0.03-0.17] | N/A | N/A | -0.29  [-0.48--0.23] | N/A | N/A | 0.07  [-0.01-0.19] | N/A |

IQR represents inter-quartile range.

θMax is maximal dorsiflexion angle for positions P1 and P2, knee flexion angle for positions P3 and P4 and hip flexion angle for position P5.

For positions P1, P2 and P4, we reported M_θMax_ values at low and high velocities.

For positions P3 and P5, we reported Mθ_Max_ are reported at low velocity only.

N/A represents unavailable data for some cases, positions and/or velocities.

|  | **M_θMin_ values (Nm/kg)**  **Median [IQR]** | | | | | | | | | | | | | | | | | |
| --- | --- | --- | --- | --- | --- | --- | --- | --- | --- | --- | --- | --- | --- | --- | --- | --- | --- | --- |
|  | Reference | | | Case A | | | Case B | | | Case C | | | Case D | | | Case E | | |
|  | LV &HV | LV | HV | LV &HV | LV | HV | LV &HV | LV | HV | LV &HV | LV | HV | LV &HV | LV | HV | LV &HV | LV | HV |
| P1 | N/A | 0.01  [0.00-0.04] | N/A | N/A | 0.02  [0.00-0.05] | N/A | N/A | 0.00  [-0.01-0.01] | N/A | N/A | -0.02  [-0.02--0.01] | N/A | N/A | 0.01  [-0.01-0.03] | N/A | N/A | N/A | N/A |
| P2 | N/A | 0.03  [0.02-0.05] | N/A | N/A | 0.03  [0.02-0.06] | N/A | N/A | -0.01  [-0.02-0.02] | N/A | N/A | -0.03  [-0.05--0.01] | N/A | N/A | 0.02  [0.01-0.04] | N/A | N/A | N/A | N/A |
| P3 | N/A | 0.03  [-0.01-0.04] | N/A | N/A | 0.03  [-0.04-0.04] | N/A | N/A | 0.03  [0.01-0.04] | N/A | N/A | 0.02  [0.00-0.04] | N/A | N/A | -0.07  [-0.1--0.04] | N/A | N/A | 0.03  [-0.01-0.04] | N/A |
| P4 | 0.16  [0.13-0.26] | 0.16  [0.12-0.20] | 0.19  [0.13-0.32] | 0.18  [0.15-0.28] | 0.18  [0.13-0.22] | 0.22  [0.15-0.37] | 0.16  [0.14-0.26] | 0.16  [0.13-0.21] | 0.21  [0.15-0.35] | 0.16  [0.14-0.24] | 0.16  [0.12-0.21] | 0.21  [0.15-0.35] | 0.20  [0.15-0.28] | 0.20  [0.17-0.27] | 0.18  [0.15-0.30] | 0.17  [0.13-0.27] | 0.17  [0.13-0.21] | 0.19  [0.13-0.32] |
| P5 | N/A | 0.64  [0.52-1.01] | N/A | N/A | 0.63  [0.52-1.02] | N/A | N/A | 0.63  [0.53-1.01] | N/A | N/A | 0.63  [0.52-0.97] | N/A | N/A | 0.29  [0.14-0.60] | N/A | N/A | 0.67  [0.58-1.03] | N/A |

**Table S2: Intersegmental moments at minimal angles for the different cases**

IQR represents inter-quartile range.

θMin is minimal dorsiflexion angle for positions P1 and P2, knee flexion angle for positions P3 and P4 and hip flexion angle for position P5.

For positions P1, P2 and P4, we reported M_θMin_ values at low velocity only.

For position P3, we reported M_θMin_ are reported at low and high velocities.

For position P5, we reported M_θMin_ values at low velocity only.

N/A represents unavailable data for some cases, positions and/or velocities.

**Table S3: Intersegmental moments differences at maximal angles for low velocity**

|  | Case A | | | Case B | | | Case C | | | Case D | | | Case E | | |
| --- | --- | --- | --- | --- | --- | --- | --- | --- | --- | --- | --- | --- | --- | --- | --- |
|  | Median [IQR] | p-value | CI 95% | Median [IQR] | p-value | CI 95% | Median [IQR] | p-value | CI 95% | Median [IQR] | p-value | CI 95% | Median [IQR] | p-value | CI 95% |
| P1 | 0.4 [-0.2-1.9] | 0.04 | [0.0-1.8] | -6.0 [-7.0--3.9] | **4e-4** | [-7.0--4.6] | -8.7 [-11.1--6.7] | **4e-4** | [-10.3--7.0] | 0.2 [-0.1-0.3] | 0.06 | [0.0-0.3] | N/A | N/A | N/A |
| P2 | 1.2 [0.8-2.5] | **4e-4** | [0.9-2.3] | -8.1 [-9.3--6.0] | **4e-4** | [-9.6--6.1] | -14.0 [-17.1--8.2] | **4e-4** | [-16.4--10.1] | 0.0 [-0.2-0.1] | 0.53 | [-0.2-0.1] | N/A | N/A | N/A |
| P3 | -1.7 [-3.3-1.3] | 0.16 | [-2.8-1.1] | 0.4 [-0.2-1.0] | 0.15 | [-0.2-0.9] | -1.7 [-4.0--0.6] | **6e-3** | [-4.5--0.6] | 8.9 [6.4-12.1] | **5e-4** | [6.4-11.5] | 0.5 [0.3-0.6] | **4e-4** | [0.3-0.6] |
| P4 | 1.2 [-0.4-4.4] | **4e-3** | [0.1-3.9] | 0.5 [-0.3-0.8] | 0.28 | [-0.4-0.7] | 0.5 [-2.3-1.9] | 0.83 | [-2.1-1.7] | 11.6 [10.4-13.0] | **5e-4** | [9.8-12.7] | 0.3 [0.1-0.4] | **6e-5** | [0.2-0.4] |
| P5 | -1.1 [-2.1-0.7] | 0.16 | [-2.1-0.5] | -0.2 [-1.4-1.0] | 0.50 | [-1.2-0.6] | 0.2 [-0.7-2.0] | 0.38 | [-0.5-1.8] | 38.7 [35.5-41.5] | **4e-4** | [35.0-44.7] | -2.1 [-2.8--1.4] | **4e-3** | [-2.8--1.1] |

IQR represents inter-quartile range, CI represents confidence interval.

All differences between cases and reference inverse dynamics methods are given in (10^-2^Nm/kg).

N/A represents unavailable data for some cases and positions.

P-values for significant differences are in bold.

**Table S4: Intersegmental moments differences at maximal angles for high velocity**

|  | Case A | | | Case B | | | Case C | | | Case D | | | Case E | | |
| --- | --- | --- | --- | --- | --- | --- | --- | --- | --- | --- | --- | --- | --- | --- | --- |
|  | Median [IQR] | p-value | CI 95% | Median [IQR] | p-value | CI 95% | Median [IQR] | p-value | CI 95% | Median [IQR] | p-value | CI 95% | Median [IQR] | p-value | CI 95% |
| P1 | 0.2 [0.5-1.4] | 0.50 | [-0.5-1.2] | -5.6 [-6.2--4.3] | **4e-4** | [-6.7--4.3] | -10.6 [-12.3--7.6] | **4e-4** | [-12.5--8.0] | 0.2 [-0.1-0.3] | 0.03 | [0.0-0.3] | N/A | N/A | N/A |
| P2 | 1.5 [0.4-2.2] | **1e-3** | [0.8-2.0] | -8.2 [-10.9--6.6] | **4e-4** | [-10.5--6.7] | -18.1 [-20.7--12.8] | **4e-4** | [-20.0--13.1] | 0.1 [0.0-0.3] | 0.19 | [-0.1-0.2] | N/A | N/A | N/A |
| P3 | -1.9 [-4.8-1.5] | 0.18 | [-4.0-1.3] | 2.1 [1.0-3.4] | **1e-3** | [1.2-3.4] | 1.4 [-0.2-3.5] | 0.13 | [-0.6-2.9] | -0.9 [-6.5-5.7] | 0.95 | [-4.9-4.2] | -0.3 [-0.8-0.2] | 0.11 | [-0.8-0.1] |
| P4 | N/A | N/A | N/A | N/A | N/A | N/A | N/A | N/A | N/A | N/A | N/A | N/A | N/A | N/A | N/A |
| P5 | N/A | N/A | N/A | N/A | N/A | N/A | N/A | N/A | N/A | N/A | N/A | N/A | N/A | N/A | N/A |

IQR represents inter-quartile range, CI represents confidence interval.

All differences between cases and reference inverse dynamics methods are given in (10^-2^Nm/kg).

N/A represents unavailable data for some cases and positions.

P-values for significant differences are in bold.

**Table S5: Intersegmental moments differences at maximal angles for low and high velocities**

|  | Case A | | | Case B | | | Case C | | | Case D | | | Case E | | |
| --- | --- | --- | --- | --- | --- | --- | --- | --- | --- | --- | --- | --- | --- | --- | --- |
|  | Median [IQR] | p-value | CI 95% | Median [IQR] | p-value | CI 95% | Median [IQR] | p-value | CI 95% | Median [IQR] | p-value | CI 95% | Median [IQR] | p-value | CI 95% |
| P1 | 0.3 [-0.3-1.8] | 0.06 | [0.0-1.2] | -5.8 [-6.7--4.3] | **7e-7** | [-6.4--4.9] | -9.4 [-11.5--6.8] | **7e-7** | [-10.8--8.2] | 0.2 [-0.1-0.3] | **4e-3** | [0.1-0.3] | N/A | N/A | N/A |
| P2 | 0.8 [0.1-2.2] | **1e-3** | [0.4-1.5] | -6.1 [-8.4--4.7] | **7e-7** | [-7.6--5.6] | -11.0 [-15.7--7.6] | **7e-7** | [-13.8--9.9] | 0.0 [-0.1-0.3] | 0.22 | [0.0-0.2] | N/A | N/A | N/A |
| P3 | -1.8 [-3.7-1.4] | 0.06 | [-2.7-0.1] | 1.0 [0.3-2.3] | **4e-4** | [0.6-1.9] | -0.5 [-3.0-1.4] | 0.45 | [-2.0-0.7] | 6.2 [1.8-11.0] | **5e-3** | [1.5-8.0] | 0.2 [-0.3-0.5] | 0.19 | [-0.1-0.3] |
| P4 | N/A | N/A | N/A | N/A | N/A | N/A | N/A | N/A | N/A | N/A | N/A | N/A | N/A | N/A | N/A |
| P5 | N/A | N/A | N/A | N/A | N/A | N/A | N/A | N/A | N/A | N/A | N/A | N/A | N/A | N/A | N/A |

IQR represents inter-quartile range, CI represents confidence interval.

All differences between cases and reference inverse dynamics methods are given in (10^-2^Nm/kg).

N/A represents unavailable data for some cases and positions.

P-values for significant differences are in bold.

**Table S6: Intersegmental moments differences at minimal angles for low velocity**

|  | Case A | | | Case B | | | Case C | | | Case D | | | Case E | | |
| --- | --- | --- | --- | --- | --- | --- | --- | --- | --- | --- | --- | --- | --- | --- | --- |
|  | Median [IQR] | p-value | CI 95% | Median [IQR] | p-value | CI 95% | Median [IQR] | p-value | CI 95% | Median [IQR] | p-value | CI 95% | Median [IQR] | p-value | CI 95% |
| P1 | -0.4 [-0.8--0.2] | 0.04 | [-0.8--0.1] | 3.0 [0.4-3.6] | **6e-4** | [1.6-3.6] | 4.3 [2.1-7.1] | **4e-4** | [2.8-5.9] | 0.8 [0.6-1.0] | **4e-4** | [0.6-0.9] | N/A | N/A | N/A |
| P2 | -1.0 [-1.6--0.3] | **4e-3** | [-1.6--0.5] | 3.6 [0.9-5.0] | **7e-4** | [1.8-4.6] | 4.9 [3.1-8.0] | **4e-4** | [3.6-8.1] | 0.7 [0.5-0.8] | **4e-4** | [0.5-0.8] | N/A | N/A | N/A |
| P3 | 0.7 [-0.4-2.7] | 0.09 | [-0.4-2.5] | -0.8 [-1.5-0.0] | **0.01** | [-1.2--0.3] | 0.3 [-0.2-1.0] | 0.25 | [-1.0-0.8] | 8.2 [7.3-10.9] | **4e-4** | [7.5-9.7] | -0.1 [-0.2-0.1] | 0.21 | [-0.2-0.1] |
| P4 | -1.2 [-2.6--0.6] | **5e-4** | [-2.3--0.7] | -0.3 [-0.6-0.4] | 0.38 | [-0.6-0.3] | -0.2 [-0.7-1.3] | 0.64 | [-0.6-1.5] | -5.9 [-7.3- -1.1] | **1e-3** | [-7.1--2.3] | -0.3 [-0.4--0.1] | **6e-5** | [-0.3--0.2] |
| P5 | -0.6 [-1.2-0.2] | 0.07 | [-1.3-0.1] | -0.1 [-0.7-1.1] | 0.80 | [-0.5-1.0] | 0.7 [-1.0-2.9] | 0.18 | [-0.4-3.0] | 34.7 [27.4-42.2] | **4e-4** | [29.0-39.4] | -4.0 [-4.6--2.6] | **4e-4** | [-4.5--2.9] |

IQR represents inter-quartile range, CI represents confidence interval.

All differences between cases and reference inverse dynamics methods are given in (10^-2^Nm/kg).

N/A represents unavailable data for some cases and positions.

P-values for significant differences are in bold.

**Table S7: Intersegmental moments differences at minimal angles for high velocity**

|  | Case A | | | Case B | | | Case C | | | Case D | | | Case E | | |
| --- | --- | --- | --- | --- | --- | --- | --- | --- | --- | --- | --- | --- | --- | --- | --- |
|  | Median [IQR] | p-value | CI 95% | Median [IQR] | p-value | CI 95% | Median [IQR] | p-value | CI 95% | Median [IQR] | p-value | CI 95% | Median [IQR] | p-value | CI 95% |
| P1 | N/A | N/A | N/A | N/A | N/A | N/A | N/A | N/A | N/A | N/A | N/A | N/A | N/A | N/A | N/A |
| P2 | N/A | N/A | N/A | N/A | N/A | N/A | N/A | N/A | N/A | N/A | N/A | N/A | N/A | N/A | N/A |
| P3 | N/A | N/A | N/A | N/A | N/A | N/A | N/A | N/A | N/A | N/A | N/A | N/A | N/A | N/A | N/A |
| P4 | -1.8 [-3.6--0.4] | **2e-3** | [-3.0--0.8] | -1.5 [-3.4--0.8] | **4e-4** | [-2.9--1.0] | -1.0 [-2.1-0.3] | 0.04 | [-2.2--0.1] | -1.1 [-2.7-2.7] | 0.75 | [-2.3-3.6] | -0.1 [-0.2-0.1] | 0.53 | [-0.2-0.2] |
| P5 | N/A | N/A | N/A | N/A | N/A | N/A | N/A | N/A | N/A | N/A | N/A | N/A | N/A | N/A | N/A |

IQR represents inter-quartile range, CI represents confidence interval.

All differences between cases and reference inverse dynamics methods are given in (10^-2^Nm/kg).

N/A represents unavailable data for some cases and positions.

P-values for significant differences are in bold.

**Table S8: Intersegmental moments differences at minimal angles for low and high velocities**

|  | Case A | | | Case B | | | Case C | | | Case D | | | Case E | | |
| --- | --- | --- | --- | --- | --- | --- | --- | --- | --- | --- | --- | --- | --- | --- | --- |
|  | Median [IQR] | p-value | CI 95% | Median [IQR] | p-value | CI 95% | Median [IQR] | p-value | CI 95% | Median [IQR] | p-value | CI 95% | Median [IQR] | p-value | CI 95% |
| P1 | N/A | N/A | N/A | N/A | N/A | N/A | N/A | N/A | N/A | N/A | N/A | N/A | N/A | N/A | N/A |
| P2 | N/A | N/A | N/A | N/A | N/A | N/A | N/A | N/A | N/A | N/A | N/A | N/A | N/A | N/A | N/A |
| P3 | N/A | N/A | N/A | N/A | N/A | N/A | N/A | N/A | N/A | N/A | N/A | N/A | N/A | N/A | N/A |
| P4 | -1.7 [-2.8--0.6] | **6e-6** | [-2.3--1.1] | -0.8 [-1.6--0.3] | **3e-4** | [-1.5--0.4] | -0.6 [-1.1-0.6] | 0.21 | [-0.9-0.3] | -2.2 [-5.9--0.1] | **9e-3** | [-4.0--0.8] | -0.2 [-0.3-0.0] | **4e-3** | [-0.2--0.1] |
| P5 | N/A | N/A | N/A | N/A | N/A | N/A | N/A | N/A | N/A | N/A | N/A | N/A | N/A | N/A | N/A |

IQR represents inter-quartile range, CI represents confidence interval.

All differences between cases and reference inverse dynamics methods are given in (10^-2^Nm/kg).

N/A represents unavailable data for some cases and positions.

P-values for significant differences are in bold.

**Table S9: Root-mean-square differences, root-mean-square differences normalized by the absolute maximum joint moment, and coefficient of correlation for low velocity**

|  | Case A (Median [IQR]) | | | Case B (Median [IQR]) | | | Case C (Median [IQR]) | | | Case D (Median [IQR]) | | | Case E (Median [IQR]) | | |
| --- | --- | --- | --- | --- | --- | --- | --- | --- | --- | --- | --- | --- | --- | --- | --- |
|  | RMSD | %Max | R | RMSD | %Max | R | RMSD | %Max | R | RMSD | %Max | R | RMSD | %Max | R |
| P1 | 0.6  [0.3-0.8] | 3.0  [2.0-4.9] | 0.99  [0.99-0.99] | 2.3  [2.1-3.3] | 15.1  [13.3-16.4] | 0.98  [0.98-0.99] | 3.6  [2.7-4.7] | 23.9  [17.7-25.5] | 0.91  [0.88-0.96] | 0.6  [0.4-0.7] | 3.6  [1.8-4.6] | 0.99  [0.99-0.99] | N/A | N/A | N/A |
| P2 | 0.8  [0.6-1.1] | 4.0  [3.1-5.8] | 0.99  [0.99-0.99] | 3.8  [2.9-4.7] | 17.7  [15.2-23.6] | 0.98  \|0.98-0.99] | 5.9  [3.8-8.1] | 30.0  [24.9-31.8] | 0.84  [0.58-0.94] | 0.4  [0.3-0.6] | 1.8  [1.3-2.5] | 0.99  [0.99-0.99] | N/A | N/A | N/A |
| P3 | 1.9  [1.4-3.0] | 5.4  [3.3-10.1] | 0.99  [0.98-0.99] | 1.0  [0.6-1.2] | 3.4  [2.1-4.5] | 0.99  [0.99-0.99] | 2.0  [1.1-3.8] | 6.4  [3.5-11.7] | 0.99  [0.97-0.99] | 10.9  [9.6-13.5] | 30.9  [21.8-52.6] | 0.98  [0.97-0.99] | 0.4  [0.3-0.4] | 0.9  [0.7-1.7] | 0.99  [0.99-0.99] |
| P4 | 1.6  [1.0-2.0] | 7.5  [4.4-15.5] | 0.99  [0.99-0.99] | 0.5  [0.4-0.6] | 2.9  [1.8-4.6] | 0.99  [0.99-0.99] | 1.3  [0.8-1.8] | 6.7  [5.3-13.1] | 0.99  [0.99-0.99] | 8.2  [7.3-9.4] | 40.1  [30.2-65.2] | 0.98  [0.96-0.98] | 0.3  [0.2-0.3] | 1.6  [0.8-1.9] | 0.99  [0.99-0.99] |
| P5 | 1.2  [0.7-1.6] | 1.1  [0.7-3.1] | 0.99  [0.99-0.99] | 1.2  [0.6-1.5] | 1.8  [0.7-2.4] | 0.99  [0.99-0.99] | 1.7  [1.3-2.2] | 2.5  [1.4-3.5] | 0.99  [0.99-0.99] | 41.1  [36.7-47.7] | 57.9  [34.3-74.1] | 0.99  [0.98-0.99] | 0.5  [0.3-0.5] | 5.8  [3.2-8.9] | 0.99  [0.99-0.99] |

IQR represents inter-quartile range, RMSD represents root-mean-square difference, %Max represents root-mean-square difference normalized by the absolute maximum joint moment, and R represents coefficient of correlation.

RMSD and %Max are given in (10^-2^Nm/kg).

N/A represents unavailable data for some cases and positions.

**Table S10: Root-mean-square differences, root-mean-square differences normalized by the absolute maximum joint moment, and coefficient of correlation for high velocity**

|  | Case A (Median [IQR]) | | | Case B (Median [IQR]) | | | Case C (Median [IQR]) | | | Case D (Median [IQR]) | | | Case E (Median [IQR]) | | |
| --- | --- | --- | --- | --- | --- | --- | --- | --- | --- | --- | --- | --- | --- | --- | --- |
|  | RMSD | %Max | R | RMSD | %Max | R | RMSD | %Max | R | RMSD | %Max | R | RMSD | %Max | R |
| P1 | 1.0  [0.4-1.3] | 4.2  [2.4-5.5] | 0.99  [0.99- | 3.4  [2.6-4.6] | 16.4  [14.6-18.4] | 0.98  [0.97-0.99] | 6.3  [4.4-8.8] | 31.9  [23.4-37.1] | 0.92  [0.70-0.96] | 0.7  [0.6-0.7] | 4.1  [1.9-5.6] | 0.99  [0.99-0.99] | N/A | N/A | N/A |
| P2 | 1.0  \|0.8-1.7] | 4.0  [3.3-6.8] | 0.99  [0.99- | 5.7  [3.5-6.8] | 21.4  [14.3-25.8] | 0.99  [0.98-0.99] | 9.9  [7.7-12.4] | 39.1  [35.1-44.3] | 0.80  [0.50-0.92] | 0.7  [0.5-0.7] | 3.2  [1.4-3.9] | 0.99  [0.99-0.99] | N/A | N/A | N/A |
| P3 | 4.1  [2.6-5.3] | 8.6  [6.9-13.1] | 0.99  [0.97- | 1.5  [1.2-2.6] | 3.8  [3.3-6.3] | 0.99  [0.99-0.99] | 2.5  [1.7-3.2] | 6.2  [3.9-8.7] | 0.98  [0.98-0.99] | 12.9  [11.3-12.9] | 35.4  [20.1-48.5] | 0.76  [0.54-0.89] | 0.6  [0.4-0.8] | 1.5  [0.9-2.1] | 0.99  [0.99-0.99] |
| P4 | 2.0  [1.0-2.5] | 5.7  [5.1-7.7] | 0.99  [0.98- | 1.7  [0.9-2.7] | 6.2  [4.0-8.8] | 0.99  [0.98-0.99] | 2.4  [1.6-3.0] | 8.4  [4.7-10.8] | 0.99  [0.97-0.99] | 7.7  [6.0-8.9] | 23.1  [17.8-33.3] | 0.83  [0.64-0.87] | 0.7  [0.4-0.8] | 1.7  [1.2-2.7] | 0.99  [0.99-0.99] |
| P5 | N/A | N/A | N/A | N/A | N/A | N/A | N/A | N/A | N/A | N/A | N/A | N/A | N/A | N/A | N/A |

IQR represents inter-quartile range, RMSD represents root-mean-square difference, %Max represents root-mean-square difference normalized by the absolute maximum joint moment, and R represents coefficient of correlation.

RMSD and %Max are given in (10^-2^Nm/kg).

N/A represents unavailable data for some cases and positions.

**Table S11: Root-mean-square differences, root-mean-square differences normalized by the absolute maximum joint moment, and coefficient of correlation for low and high velocities**

|  | Case A (Median [IQR]) | | | Case B (Median [IQR]) | | | Case C (Median [IQR]) | | | Case D (Median [IQR]) | | | Case E (Median [IQR]) | | |
| --- | --- | --- | --- | --- | --- | --- | --- | --- | --- | --- | --- | --- | --- | --- | --- |
|  | RMSD | %Max | R | RMSD | %Max | R | RMSD | %Max | R | RMSD | %Max | R | RMSD | %Max | R |
| P1 | 0.7  [0.4-1.1] | 3.4  [2.4-5.0] | 0.99  [0.99-0.99] | 3.0  [2.1-3.8] | 15.8  [14.0-17.2] | 0.98  [0.97-0.99] | 4.7  [3.3-6.5] | 24.8  [21.9-31.9] | 0.92  [0.84-0.95] | 0.6  [0.4-0.8] | 3.8  [1.9-5.4] | 0.99  [0.99-0.99] | N/A | N/A | N/A |
| P2 | 1.0  [0.7-1.6] | 4.1  [3.3-6.5] | 0.99  [0.99-0.99] | 4.3  [3.1-5.8] | 19.6  [14.3-24.1] | 0.99  [0.97-0.99] | 7.8  [5.3-10.8] | 32.0  [28.3-40.0] | 0.82  [0.58-0.92] | 0.5  [0.4-0.7] | 1.9  [1.3-3.6] | 0.99  [0.99-0.99] | N/A | N/A | N/A |
| P3 | 2.9  [1.8-4.2] | 7.6  [4.5-12.0] | 0.99  [0.98-0.99] | 1.2  [0.9-1.8] | 3.6  [2.9-4.7] | 0.99  [0.99-0.99] | 2.1  [1.4-3.2] | 6.3  [3.8-9.4] | 0.99  [0.97-0.99] | 11.8  [10.4-14.1] | 34.2  [21.3-50.4] | 0.95  [0.76-0.98] | 0.4  [0.3-0.6] | 1.4  [0.8-1.8] | 0.99  [0.99-0.99] |
| P4 | 1.7  [1.0-2.4] | 6.1  [5.1-10.4] | 0.99  [0.98-0.99] | 0.9  [0.4-1.7] | 4.5  [2.5-7.5] | 0.99  [0.99-0.99] | 1.6  [1.2-2.7] | 8.0  [4.8-12.1] | 0.99  [0.98-0.99] | 8.0  [6.9-9.3] | 31.1  [19.9-47.3] | 0.92  [0.82-0.98] | 0.3  [0.2-0.7] | 1.6  [1.1-2.5] | 0.99  [0.99-0.99] |
| P5 | N/A | N/A | N/A | N/A | N/A | N/A | N/A | N/A | N/A | N/A | N/A | N/A | N/A | N/A | N/A |

IQR represents inter-quartile range, RMSD represents root-mean-square difference**,** %Max represents root-mean-square difference normalized by the absolute maximum joint moment, and R represents coefficient of correlation.

RMSD and %Max are given in (10^-2^Nm/kg).

N/A represents unavailable data for some cases and positions.

**Figure S1 – Measured forces and moments during the different passive mobilizations**

This supplementary material presents several graphics of the measured forces and moments during the different passive mobilizations.

These results show that when applying a stretch, the evaluator does not apply a purely monoaxial force (along the Z-axis), but that tangential forces and moments are also present.

Figure S1 – A, proposed for indicative purpose, shows the dynamometer frame under the several positions.

Figure S1 – A: Test positions. Dynamometer axis are in red.

**
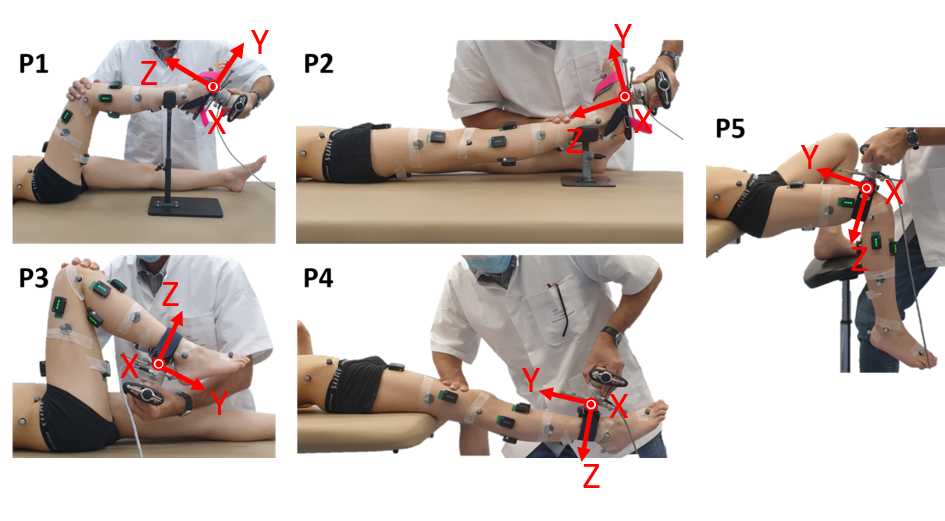
**

Each figure below shows measured forces and moments during the different passive mobilizations for all the subjects. For each trial, the time is normalized by the duration of the trial and the studied joint is mobilized three times.

Figure S1 – B: Measured forces and moments of all the subjects in position P1 at low and high velocity.


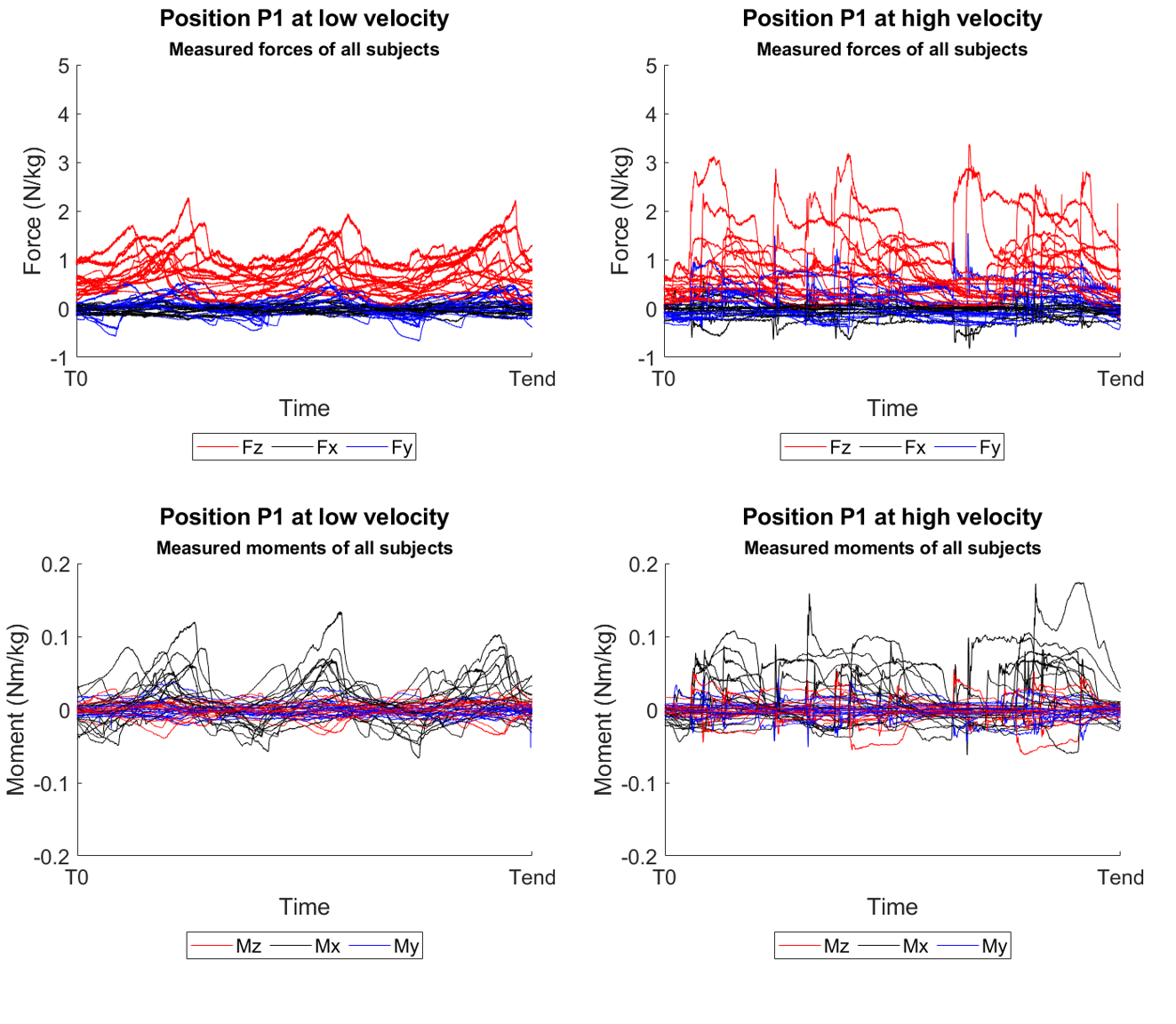


Figure S1 – C: Measured forces and moments of all the subjects in position P2 at low and high velocity.


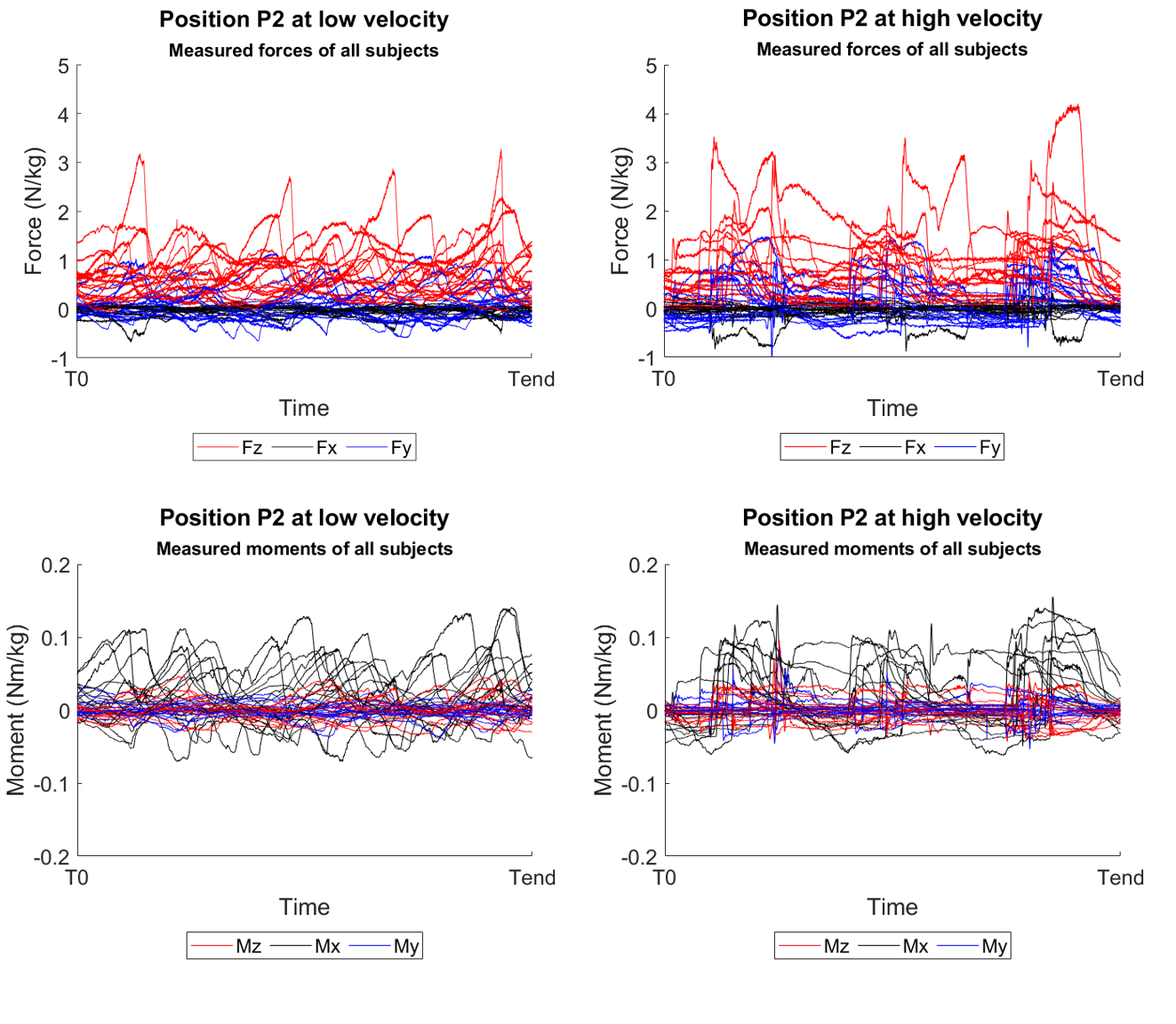


Figure S1 – D: Measured forces and moments of all the subjects in position P3 at low and high velocity.


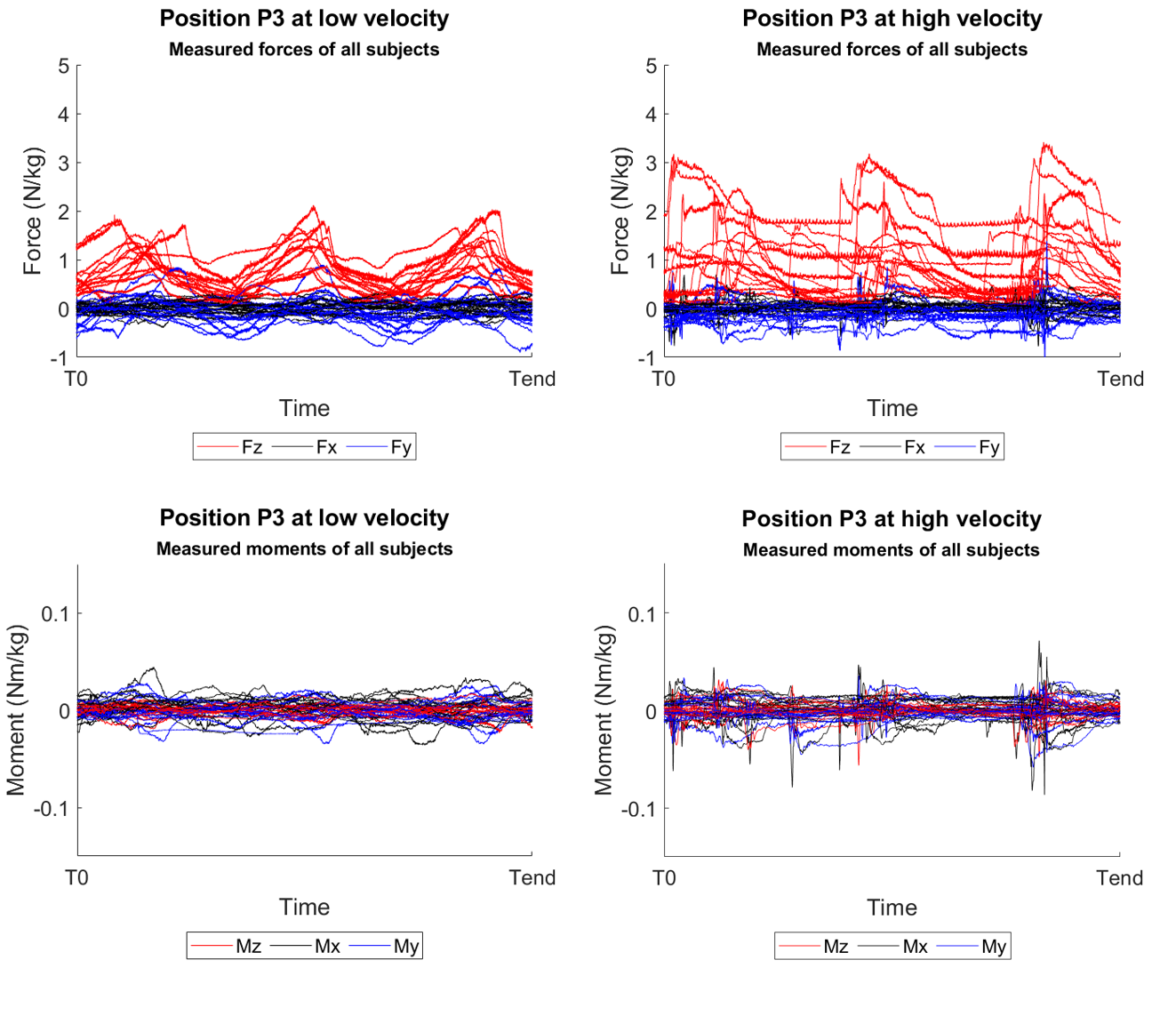


Figure S1 – E: Measured forces and moments of all the subjects in position P4 at low and high velocity.


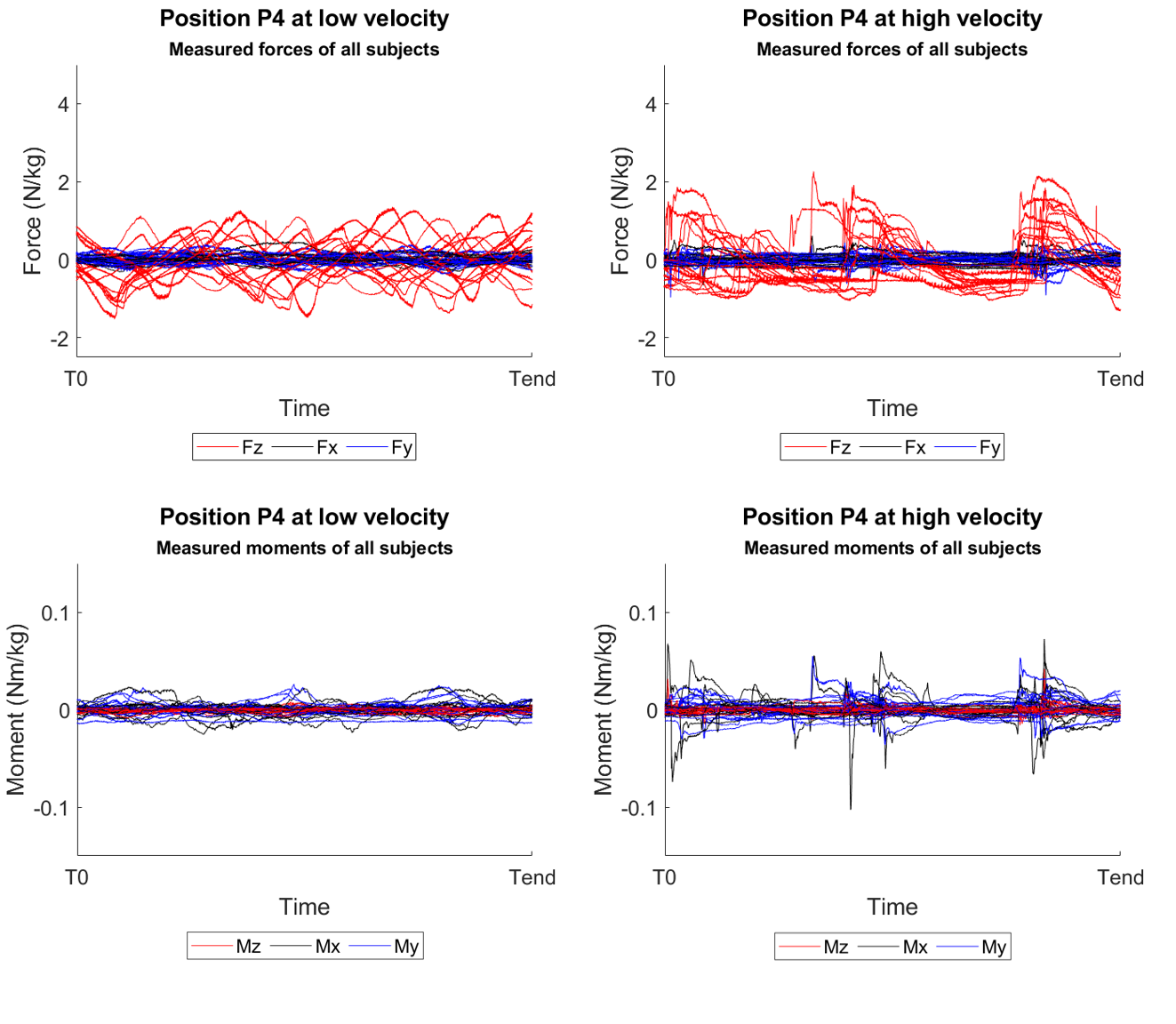


Figure S1 – F: Measured forces and moments of all the subjects in position P5 at low velocity.


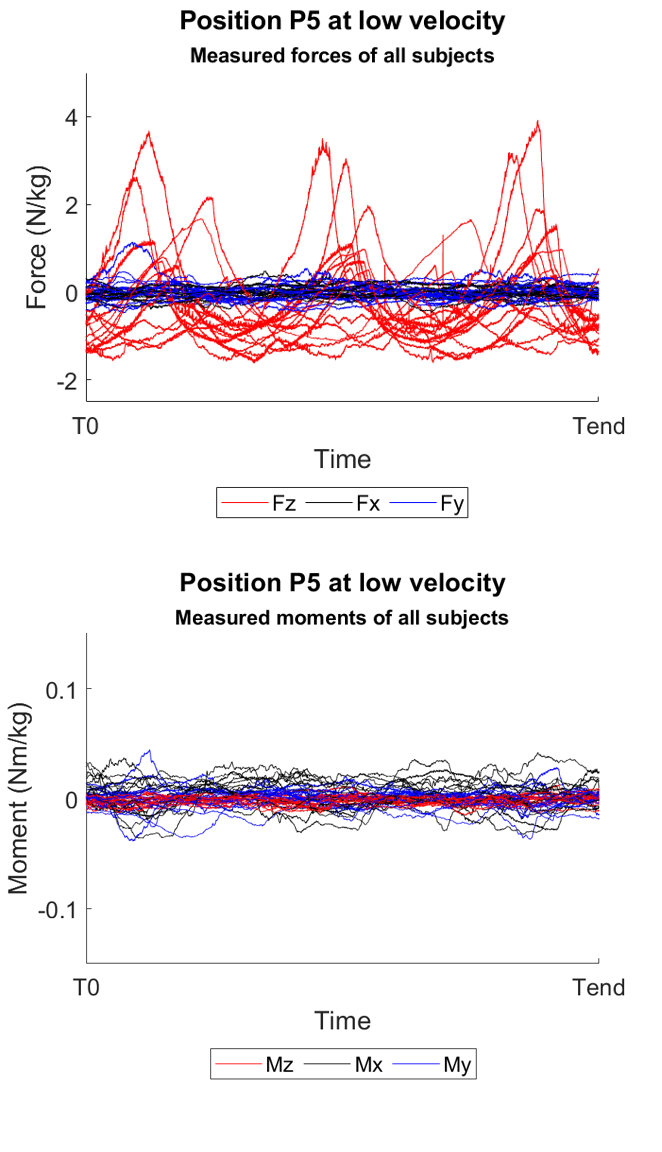

Supplement: Supplementary file 1 — Supplementary Information. [file 41598_2023_44576_MOESM1_ESM.docx]
